# Influenza-Associated Hospitalizations, Singapore, 2004–2008 and 2010–2012

**DOI:** 10.3201/eid2010.131768

**Published:** 2014-10

**Authors:** Li Wei Ang, Cindy Lim, Vernon Jian Ming Lee, Stefan Ma, Wei Wei Tiong, Peng Lim Ooi, Raymond Tzer Pin Lin, Lyn James, Jeffery Cutter

**Affiliations:** Ministry of Health, Singapore (L.W. Ang, C. Lim, V.J.M. Lee, S. Ma, W.W. Tiong, P.L. Ooi, R.T.P. Lin, L. James, J. Cutter);; Ministry of Defence, Singapore (V.J.M. Lee)

**Keywords:** vaccination, surveillance, disease burden, morbidity, influenza, pneumonia, Singapore, hospitalizations, viruses, public health policies, tropical settings, excess hospitalizations, influenza-associated

## Abstract

Studies of influenza-associated hospitalizations in tropical settings are lacking. To increase understanding of the effect of influenza in Singapore, we estimated the age-specific influenza-associated hospitalizations for pneumonia and influenza during 2004–2008 and 2010–2012. The rate of hospitalization was 28.3/100,000 person-years during 2004–2008 and 29.6/100,000 person-years during 2010–2012. The age-specific influenza-associated hospitalization rates followed a J-shaped pattern: rates in persons >75 years of age and in children <6 months of age were >47 times and >26 times higher, respectively, than those for persons 25–44 years of age. Across all ages during these 2 study periods, ≈12% of the hospitalizations for pneumonia and influenza were attributable to influenza. The rates and proportions of hospitalizations for influenza, particularly among the very young and the elderly, are considerable in Singapore and highlight the importance of vaccination in protecting populations at risk.

## Abstract 2

Vaccination helps reduce disease burden, particularly in the elderly, who are at higher risk for hospitalization and death.

Seasonal influenza causes a substantial burden of illness worldwide. Infections can lead to severe illness that requires hospital care and can occasionally lead to death. Several studies have documented influenza-associated hospitalizations in countries with primarily temperate climates, such as the United States (*1*–*5*), and others have documented influenza-associated hospitalizations in subtropical regions, mostly in Hong Kong, China (*6*–*8*).

In the tropics, the spread of influenza is different from that in temperate regions because of the unique tropical climate and lack of clear climatic seasons (*9*,*10*). The baseline incidence of influenza infection is high, and >1 seasonal epidemic occurs each year (*11*). As documented in studies on influenza-associated deaths in Singapore (*9*,*12*), the effect of influenza epidemics in the tropics is comparable to its effect in other climatic regions. However, studies on influenza-associated hospitalizations in tropical settings are lacking. Such studies can provide an understanding of the pattern of hospitalizations and severe illness that is valuable in guiding public health policies.

Laboratory testing of specimens and virologic confirmation of influenza virus infections are not typically conducted for all patients and deaths; thus, the estimation of illness attributable to influenza cannot be based on reported episodes alone. Influenza can precipitate or exacerbate other respiratory and circulatory conditions, and there is a wide spectrum of clinical pathways and outcomes for influenza-associated conditions and complications. Pneumonia was ranked fifth in the list of top 10 conditions for hospitalization in Singapore in 2012 (*13*). There is a need to estimate the effect of influenza on health care utilization in terms of hospitalization for pneumonia and influenza.

Singapore has a robust data collection system, which facilitates the integration of databases from virologic surveillance for influenza and hospital systems. The aim of our study was to examine the influenza-associated hospitalization rates and proportions of pneumonia and influenza hospitalizations in Singapore. Age groups spanning <6 months to ≥75 years of age were examined to further identify the populations at greatest risk for influenza-associated hospitalizations.

## Materials and Methods

### Data

Singapore is a tropical city-state; the 2012 mid-year population was ≈5.3 million (*14*). Inpatient information from all hospitals in Singapore is captured in electronic medical records that include discharge diagnoses based on the 9th and 10th revisions of the International Classification of Diseases (ICD).

We obtained the weekly number of hospital admissions for principal discharge diagnosis of pneumonia and influenza (ICD-9 480–487 and ICD-10 J10–J18) during 2004–2008 and 2010–2012. We excluded data for 2009 because during the influenza A(H1N1)pdm09 pandemic that year, many persons were hospitalized for isolation purposes rather than on the basis of the clinical severity of illness. Ten groupings, by age, were considered: <6 months, 6–23 months, 2–4 years, 5–14 years, 15–24 years, 25–44 years, 45–64 years, 65–74 years, >75 years, and all ages.

The Ministry of Health (MOH), Singapore, has a national surveillance program for influenza, which was enhanced after the influenza A(H1N1)pdm09 pandemic. Before epidemiologic week 22 in 2009, virologic surveillance was based on diagnostic respiratory specimens of outpatients and inpatients from public acute-care hospitals, and subtyping was conducted on selected specimens. These specimens were tested either with informed consent from patients for diagnostic purposes or as part of epidemiologic surveillance provided for by the Infectious Diseases Act (*12*). Influenza viruses were identified by direct antigen detection using immunofluorescence techniques, serologic tests with complement fixation, and virus isolation. Beginning in epidemiologic week 22 in 2009, the specimens were obtained only from outpatients with influenza-like illness (ILI), and all influenza virus–positive specimens were subtyped. Under the revamped sentinel surveillance program, nasopharyngeal and/or throat swab specimens were obtained from outpatients with ILI (temperature >38°C plus cough or sore throat) at government primary care clinics and private general practitioner clinics for influenza virus subtyping. Real-time reverse transcription PCR was used to determine influenza virus types and subtypes. Because of the change in surveillance sampling, we separately analyzed pre-2009 and post-2009 data.

### Statistical Analysis

We chose the negative binomial regression model over the Poisson regression model after using a likelihood ratio test to test the model assumption; the results demonstrated that the negative binomial regression model was a better fit with the data. For each age group and the all-age group, we fitted the following negative binomial regression model to the weekly number of hospital admissions for pneumonia and influenza: weekly number of hospitalizations = long term trend and seasonality + influenza + respiratory syncytial virus (RSV) + weekly mean temperature + weekly mean relative humidity

To estimate the effect of influenza, we entered the weekly proportions of influenza virus–positive specimens across all ages; these data were derived from virologic surveillance and comprised reports of influenza A(H3N2), seasonal influenza A(H1N1), A(H1N1)pdm09, and influenza B infections. In our models, we used the all-age rather than age-specific proportion of influenza-positive specimens because the latter would have resulted in too few specimens for statistical analysis from patients <5 and >65 years of age. In addition, laboratory testing could be skewed toward particular age groups (mainly adults), which could result in a poor fit with the observed data in underrepresented age groups.

Seasonal peaks in hospitalizations for pneumonia and influenza may also be attributable to RSV. Thus, to avoid overestimation of hospitalizations for pneumonia and influenza attributable to influenza, we included in our model the weekly proportion of diagnostic specimens with test results positive for RSV. The data on RSV were from 2 public acute-care hospitals with pediatric departments that routinely test for RSV, which is known to predominately affect young children. These 2 public acute-care hospitals covered ≈63%–68% of hospitalizations for children <15 years of age in Singapore.

We made adjustment for potential confounding by including meteorologic variables in the regression models. To control for long-term trend and seasonality, we used a natural cubic spline function (piecewise smoothing polynomials) for time. We also used a nonlinear function with a natural cubic spline for weekly mean temperature and weekly mean relative humidity.

We evaluated model validity by plotting partial autocorrelation functions, which indicated that the specifications of the studied models were adequate and, hence, autoregressive terms of residuals were not included. We used the Spearman rank correlation coefficient to compare the association between the weekly number of hospitalizations for pneumonia and influenza and the proportions of specimens with test results positive for influenza virus in the 2 study periods.

### Influenza-Associated Hospitalizations

The number of influenza-associated hospitalizations for pneumonia and influenza was defined as the sum of differences between the observed and expected weekly hospitalization numbers for pneumonia and influenza when influenza proportions were set to zero in the model (i.e., excess number attributable to influenza). We estimated the proportion of influenza-associated hospitalizations for pneumonia and influenza by dividing the total number of excess hospitalizations by the total number of observed hospitalizations.

The 95% CI for each estimated proportion was obtained by using the bootstrap resampling method with 1,000 resamples. The 2.5% and 97.5% quantiles of the 1,000 estimates were taken as the lower and upper bounds, respectively. The 95% CI for the number of influenza-associated hospitalizations for pneumonia and influenza was then derived by multiplying the number of observed hospitalizations for pneumonia and influenza by the respective 95% CI for the proportion of influenza-associated hospitalizations. The influenza-associated hospitalization rate per 100,000 person-years was obtained by dividing the total number of excess hospitalizations for pneumonia and influenza by the sum of the annual mid-year population estimates in the entire study period. The R statistical package, v3.0.0 (http://cran.r-project.org/bin/windows/base/old/3.0.0/) was used for analysis.

## Results

During 2004–2008, a total of 59,519 diagnostic specimens were tested for influenza virus (Table 1). Over this 5-year period, there was an upward trend in the annual number of specimens tested, increasing from 13.1% of the total specimens in 2004 to 28.4% of the total specimens in 2008. A total of 3,131 (5.3%) specimens were positive for influenza virus. Among the influenza virus–positive specimens, a mean annual proportion of 72.2% (range 57.0%–86.4%) were positive for influenza A and 27.8% (range 13.6%–43.0%) for influenza B.

**Table 1:** Virologic surveillance for influenza in Singapore during 2 study periods, 2004–2008 and 2010–2012

Study period	Total no. influenza–positive specimens/total no. tested	% Influenza–positive specimens	% Influenza A among influenza–positive specimens*	% Influenza B among influenza–positive specimens
Study period 1				
2004	294/7,783	3.8	67.0	33.0
2005	462/10,441	4.4	86.4	13.6
2006	535/11,105	4.4	72.9	27.1
2007	597/13,267	4.5	57.0	43.0
2008	1,243/16,923	7.3	77.6	22.4
Study period 2				
2010	3,461/6,971	49.6	78.1	21.9
2011	1,182/2,903	40.7	76.3	23.7
2012	975/2,112	46.2	52.0	48.0
*Includes all influenza A subtypes.				

During 2010–2012, a total of 11,986 specimens from outpatients with ILI were tested for influenza virus. Over this 3-year period, there was a decreasing trend in the annual number of specimens tested; 58.2% of the total specimens were tested in 2010, compared with 17.6% in 2012. A total of 5,618 (46.9%) specimens were positive for influenza virus. Among the influenza virus–positive specimens, a mean annual proportion of 68.8% (range 52.0%–78.1%) were positive for influenza A virus, compared with 31.2% (range 21.9%–48.0%) for influenza B virus.

**Figure 1:**
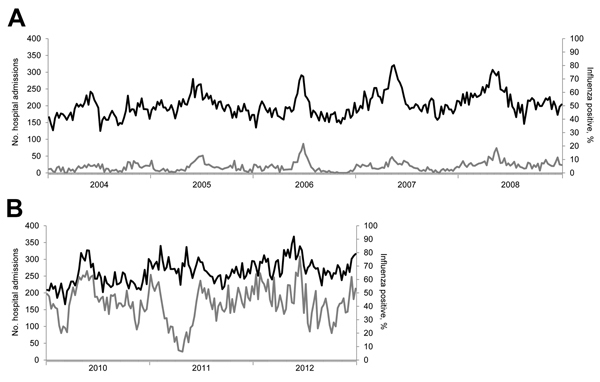
Weekly number of hospitalizations for pneumonia and influenza and proportion of influenza-positive specimens as determined on the basis of virologic surveillance, Singapore, 2004–2008 and 2010–2012. A) Hospitalizations and diagnostic specimens for 2004–2008. B) Hospitalizations and specimens for outpatients with influenza-like illness, 2010–2012. Black lines indicate hospital admissions for pneumonia and influenza; gray lines indicate % positive for influenza.

The higher percentage of influenza virus–positive specimens obtained during the second study period (46.9%), compared with percentage obtained during the first study period (5.3%), was due to the use of a more specific ILI definition for virologic surveillance and a more sensitive diagnostic method (i.e., reverse transcription PCR) during 2010–2012. For the first study period, the weekly number of hospitalizations for pneumonia and influenza and the proportion of influenza-positive diagnostic specimens were significantly correlated (p<0.01) (Figure 1). Likewise, for the second study period, the weekly number of hospitalizations for pneumonia and influenza and the proportion of influenza-positive specimens from outpatients with ILI were also significantly correlated (p<0.01) (Figure 1). The Spearman rank correlation coefficient was 0.587 during the first study period, compared with 0.314 during the second study period, when surveillance sampling was changed. These differences in correlation could be partly due to patient settings: during the first period, the specimens were obtained from patients in public acute-care hospitals, whereas during the second period, specimens were obtained from outpatients with ILI in community settings.

For all study years, except 2007, 2008, and 2011, the age-specific hospitalization rate per 100,000 person-years for pneumonia and influenza was lowest in the 25- to 44-year-old age group; for 2007, 2008, and 2011, the lowest rate was in the 15- to 24-year-old age group (Table 2). The annual hospitalization rate was consistently highest for persons >75 years of age.

**Table 2:** Age-specific hospitalization rates (per 100,000 person-years) for pneumonia and influenza in Singapore during 2 study periods, 2004–2008 and 2010–2012

	Hospitalization rates per 100,000 person-years								
Age group	Study period 1						Study period 2		
	2004	2005	2006	2007	2008		2010	2011	2012
0–5 m	531.9	514.9	437.3	720.6	813.9		791.4	1107.4	751.8
6–23 m	609.3	691.9	516.1	618.3	721.3		678.9	804.4	671.8
2–4 y	727.8	803.2	564.3	676.5	702.6		619.7	800.0	678.2
5–14 y	127.9	186.5	93.8	115.2	140.8		129.2	171.3	173.0
15–24 y	52.1	59.0	49.0	48.1	43.0		49.4	44.2	51.6
25–44 y	46.6	49.2	48.9	49.5	52.5		43.4	46.9	49.5
45–64 y	153.5	164.5	159.8	179.6	172.4		191.9	191.7	207.7
65–74 y	773.5	781.2	746.8	803.6	793.2		787.2	827.8	894.4
≥75 y	3,310.1	3,481.5	3,319.3	3,678.0	3,593.4		3,487.8	3,765.7	3,784.9
All	226.3	248.8	220.6	245.1	243.7		297.9	327.3	334.3

**Figure 2:**
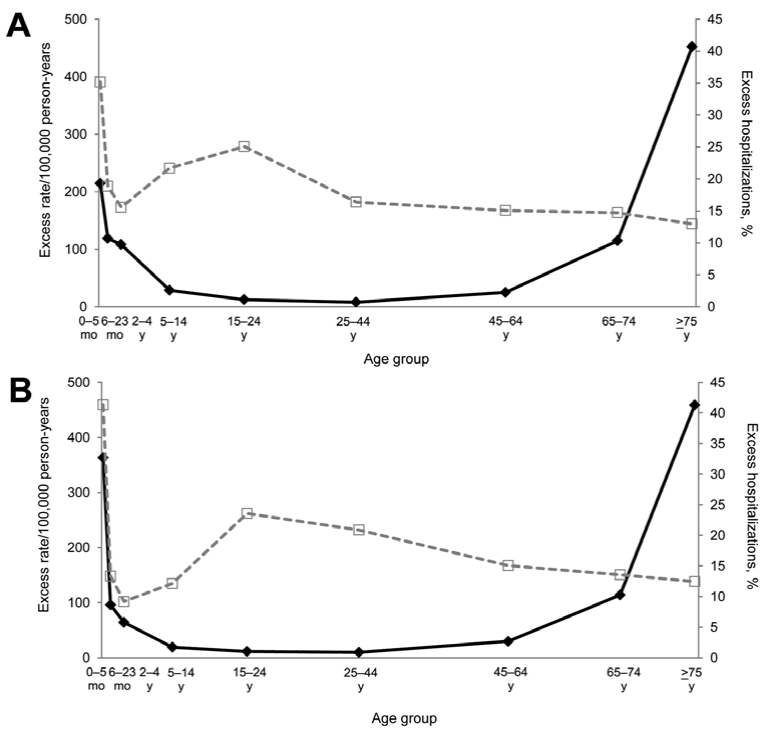
Age-specific rates (per100,000 person-years) and proportions of influenza-associated hospitalizations for pneumonia and influenza, Singapore. A) 2004–2008 and B) 2010–2012. Black bold lines indicate influenza-associated hospitalization rate per 100,000 person-years; gray dashed lines indicate % of influenza-associated hospitalizations.

In the 2 study periods, the age-specific influenza-associated hospitalization rates per 100,000 person-years for pneumonia and influenza showed a J-shaped pattern (Figure 2). The influenza-associated proportion was highest in children <6 months of age, and it was second highest in adolescents and young adults in the 15- to 24-year-old age group, followed by a decline in the older age groups in each of the 2 study periods.

During both study periods, the influenza-associated hospitalization rate per 100,000 person-years for pneumonia and influenza was highest among persons >75 years of age (452.2 and 458.9 during 2004–2008 and 2010–2012, respectively), second highest among children <6 months of age (213.9 and 363.6 during 2004–2008 and 2010–2012, respectively), and lowest among persons 25–44 years of age (8.1 and 9.7 during 2004–2008 and 2010–2012, respectively) (Table 3). The proportion of influenza-associated hospitalizations for pneumonia and influenza was highest among children <6 months of age (35.2% and 41.3% during 2004–2008 and 2010–2012, respectively). The proportion among persons 15–24 years of age was 25.1% during 2004–2008 and 23.6% during 2010–2012. The overall influenza-associated hospitalization rates per 100,000 person-years (≈30) and proportions (12%) were similar during the 2 study periods.

**Table 3:** Estimated influenza-associated hospitalizations for pneumonia and influenza in Singapore during 2 study periods, 2004–2008 and 2010–2012

Study period, patient age	Excess % (95% CI)	Excess no. per year (95% CI)	Excess no. per 100,000 person-years (95% CI)
Study period 1, 2004–2008			
0–5 m	35.2 (27.3–41.8)	40 (31–48)	213.9 (166.1–253.9)
6–23 m	18.9 (14.7–23.7)	74 (58–93)	119.1 (93.0–149.6)
2–4 y	15.6 (12.2–19.8)	141 (110–179)	108.3 (85.0–137.9)
5–14 y	21.7 (17.3–26.5)	150 (119–183)	28.8 (23.0–35.3)
15–24 y	25.1 (21.2–29.9)	87 (73–103)	12.5 (10.6–14.9)
25–44 y	16.4 (13.4–20.0)	139 (113–169)	8.1 (6.6–9.9)
45–64 y	15.1 (12.5–18.2)	251 (208–302)	25.1 (20.8–30.2)
65–74 y	14.7 (12.3–18.0)	238 (198–291)	114.9 (95.8–140.5)
≥75 y	13.0 (10.9–15.2)	518 (436–605)	452.2 (380.8–528.8)
All*	11.9 (10.4–13.8)	1,259 (1,100–1,456)	28.3 (24.7–32.7)
Study period 2, 2010–2012			
0–5 m	41.3 (25.6–56.9)	68 (42–94)	363.6 (225.0–500.5)
6–23 m	13.3 (6.7–23.4)	62 (31–108)	95.8 (48.3–168.3)
2–4 y	9.2 (4.6–16.7)	88 (44–160)	64.3 (32.4–117.2)
5–14 y	12.1 (6.0–21.5)	94 (47–166)	19.1 (9.5–33.8)
15–24 y	23.6 (12.7–38.2)	90 (49–146)	11.4 (6.2–18.5)
25–44 y	20.9 (12.3–30.5)	201 (118–294)	9.7 (5.7–14.2)
45–64 y	15.1 (9.1–22.0)	366 (220–535)	29.7 (17.9–43.5)
65–74 y	13.6 (8.2–19.9)	279 (168–409)	113.8 (68.6–167.0)
≥75 y	12.5 (7.8–18.0)	687 (431–993)	458.9 (288.3–663.8)
All*	11.2 (7.5–15.4)	1,535 (1,020–2,106)	29.6 (19.7–40.6)
*The excess number of hospitalizations was estimated from a model developed for each of the age groups and the all-age group. Thus, the number of excess hospitalizations in the all-age group was not the sum of excess hospitalizations across the 9 age groups.			

The weekly proportion of diagnostic specimens with positive test results for RSV was statistically significant in the regression models for 2 age groups (<6 months and 6–23 months of age) in the first study period and 1 age group (5–14 years of age) in the second study period (p<0.05 in these 3 models). Sensitivity analyses showed that the estimates of influenza-associated hospitalization rates and proportions with and without RSV in regression models for all the age groups varied within 2%.

## Discussion

Our analyses show that in Singapore, the age-specific influenza-associated hospitalization rates for pneumonia and influenza followed a J-shaped pattern that was also seen in analyses from other studies (*1*,*7*). The influenza-associated hospitalization rate was highest among persons >75 years of age, followed by children <6 months of age.

Our estimates of the number of influenza-associated hospitalizations in Singapore reflect the effect of influenza on the health care system in this tropical setting. The average length of hospital stay for pneumonia and influenza was ≈8 days. The mean annual estimate of influenza-associated hospitalizations for pneumonia and influenza during 2010–2012 was 1,535 (Table 3), which translates to 12,280 patient-days and a daily average of 34 occupied beds. We estimated that an additional 20 beds were occupied each day when influenza activity was at its peak. In the United States, studies estimating the national number of cases and hospitalizations averted by influenza vaccination support the use of influenza vaccination as a central tool for preventing influenza, and they highlight the need for increasing vaccination coverage and the need for more effective vaccines (*15*,*16*).

In Singapore, influenza vaccination is a key strategy for reducing the use of influenza-associated hospital services and influenza-associated illness and death. The Singapore MOH Expert Committee on Immunization recommended the use of influenza vaccine to protect vulnerable populations at higher risk for influenza-related complications; these populations include persons >65 years of age, adults and children with chronic medical conditions, pregnant women, and children 6 months to <5 years of age. Influenza vaccination has been shown to be cost-effective among the elderly and those with chronic medical conditions (*17*–*21*). Because the risk for influenza-associated hospitalization and death is high among the elderly and there may be lower effectiveness of influenza vaccine in the elderly (*22*–*25*), vaccination of household members of persons >65 years of age is encouraged to reduce transmission of influenza virus within the household (*26*–*30*).

In our study, children <6 months of age had the second highest age-specific influenza-associated hospitalization rate for pneumonia and influenza. Studies in different geographic regions (e.g., Finland, Hong Kong, and the United States) also found high hospitalization rates associated with influenza in children <1 year of age (*6*,*31*–*36*). Influenza vaccine is licensed only for use in persons >6 months of age. One way to reduce the risk of influenza in those <6 months of age is vaccination of their household contacts and caregivers, as recommended by the Advisory Committee on Immunization Practices in the United States (*30*).

Our findings underscore the importance of vaccination against influenza virus, in particular for the elderly, who are at higher risk for hospitalization and death. The Health Behavior Surveillance of Singapore, conducted by the Health Promotion Board by telephone and face-to-face with selected Singapore residents 18–69 years of age, showed that the proportion of persons who reported having been vaccinated against influenza in the preceding 12 months decreased significantly from 15.8% in 2010 to 11.2% in 2012 (p<0.001) (Health Promotion Board, unpub. data). In the 2012 Health Behavior Surveillance of Singapore, only 8.7% of adults 50–69 years of age reported having been vaccinated against influenza; this vaccine uptake percentage was half that for young adults 18–29 years of age (16.9%). In Hong Kong during the 2012–13 influenza season, vaccination coverage in the general population was 14.0%; the proportion of vaccinated persons was highest among persons >65 years of age (39.1%) and second highest among children 6 months to 5 years of age (28.4%) (*37*). In the United States during the 2011–12 influenza season 44.3% of children 6–23 months of age received full vaccination, and 38.3% of persons >18 years of age were vaccinated (*38*). The National Health Interview Survey in the United States showed an increase in influenza vaccination coverage by age: 26.1% coverage among persons 18–49 years of age, 44.0% in persons 50–64 years of age, and 69.4% in persons >65 years of age.

Various measures have been implemented to raise awareness and increase influenza vaccine uptake in Singapore. For example, since 2014, the use of Medisave, a compulsory national health care savings scheme, has been allowed for payment of seasonal influenza vaccination in populations at high risk for influenza-associated hospitalization or death, including children 6 months to <5 years of age and persons >65 years of age. In addition, the Health Promotion Board and health care providers have made consistent efforts to educate the public on the importance of influenza vaccinations.

In Singapore, the proportion of influenza-associated hospitalizations for pneumonia and influenza was highest in children <6 months of age and second highest in the 15- to 24-year-old age group. There are many factors that may have affected this pattern; for example, hospitalizations for pneumonia and influenza attributable to viruses other than influenza, and the rate of testing by age group may have had an effect (Figure 2). In addition, there may be a higher propensity for testing and hospitalizing children <6 months of age compared with persons in older age groups.

This study in Singapore provides population-based estimates of influenza-associated hospitalizations for pneumonia and influenza, which enables comparison with estimates for other countries. In Singapore, the overall influenza-associated hospitalization rate per 100,000 person-years for pneumonia and influenza was 28.3 during 2004–2008 and 29.6 during 2010–2012. The proportion of influenza-associated hospitalizations was 11.9% and 11.2%, respectively, for the same years. A similar study in Hong Kong (a subtropical location), using Poisson regression based on data from 1996–2000, estimated that the influenza-associated hospitalization rate per 100,000 person-years for all ages was 29.3 and that 11.6% of all hospitalizations for pneumonia and influenza were attributable to influenza (*7*). In a study in the United States during 1979–2001, the overall rate of influenza-associated hospitalizations per 100,000 person-years for pneumonia and influenza was 36.8, and the proportion of influenza-associated hospitalizations was 8.6% (*1*).

In addition, during the 2 study periods (2004–2008 and 2010–2012) in our study, the rates and proportions of influenza-associated hospitalizations for pneumonia and influenza among the elderly (Table 3) were higher than those in Hong Kong during 1996–2000. In Hong Kong, the influenza-associated hospitalization rate per 100,000 person-years was 58.7 (95% CI 43.3–73.7) for persons 65–74 years of age and 176.3 (95% CI 119.2–231.0) for persons >75 years of age; the influenza-associated proportion was 11.0 (95% CI 8.1–13.8) and 7.1 (95% CI 4.8–9.3) in these 2 age groups, respectively (*7*). In Singapore, the influenza-associated hospitalization rate per 100,000 person-years among children <5 years of age was 92.3 (95% CI 74.1–118.3) during 2004–2008 and 65.8 (95% CI 36.5–114.6) during 2010–2012 (results not shown); these rates far exceeded the rate of 18.5 in the same age group in the United States during 1979–2001 (*1*). Various factors must be considered when comparing influenza-associated hospitalization rates and proportions by country. For example, comparisons should consider the age structure of the populations; influenza vaccination coverage, the level of herd immunity, and access to and utilization of health care services within the communities; the socioeconomic profile of the communities; and the climatic variables and severity of influenza epidemics in the communities. There are also variations in the statistical models, data aggregation, study periods, sampling protocols, and the coverage of laboratory virus surveillance systems.

Our study has several limitations. First, influenza-associated hospital admissions for pneumonia and influenza could be affected by many factors, such as uptake of influenza vaccines in the population, dominant influenza virus types/subtypes and their antigenic drifts and shifts, seasonal variations in vaccine match to circulating influenza strains, changes in admission criteria and diagnostic practices in hospitals, and variations in health care–seeking behavior. Second, although we controlled for potential confounding factors in our modeling approach, there may still be several unmeasured factors that could affect the estimates of influenza-associated hospitalizations for pneumonia and influenza. Third, in 2012 in Singapore, the diagnosis coding system for hospitals changed from ICD-9 to ICD-10; this change could have complicated the practice and assessment of cause-specific diagnoses. Last, our analysis was confined to hospitalizations for which the principal discharge diagnoses were pneumonia and influenza. However, during influenza seasons hospitalization rates increased for conditions other than pneumonia (e.g., acute bronchitis, chronic respiratory disease, and congestive heart failure) (*39*). In our study, we did not estimate the effect of hospitalizations for these other influenza-attributable conditions.

Our findings allude to the importance of surveillance data for monitoring the effect of influenza and for assessing changes in influenza dynamics, as determined on the basis of a well-integrated virologic and epidemiologic surveillance system. For 2 reasons, we assumed that variation in influenza virologic surveillance by the MOH accurately reflected the patterns of influenza circulation in the general Singaporean population of Singapore: 1) during 2004–2008, the diagnostic respiratory specimens we used were from all public acute-care hospitals, and 2) the specimens during 2010–2012 were from outpatients with ILI at government primary care clinics and private general practitioner clinics that were geographically spread out across Singapore. In Singapore, 80% of the primary health care services are provided by private general practitioners; government clinics provide the remaining 20% of the primary health care services. The opposite is true for hospitalization care: public sector hospitalizations constitute 80% of all hospital admissions, and private sector hospitalizations constitute the remaining 20% (*40*). However, over time, there could be variation in laboratory testing patterns and sentinel sites for specimen collection in the community. Although we were unable to assess the effect of this variation, which may be somewhat reflected in the annual number of specimens tested, our modeling study was supported by sufficient year-round data for virologic surveillance.

Our findings have obvious policy implications. The J-shaped pattern observed for influenza-associated hospitalization rates during the 2 study periods was also seen in all of the individual years, both before and after the influenza A(H1N1)pdm09 pandemic. Our findings also underscore the importance of continuous surveillance in Singapore to identify populations at high risk for influenza-associated hospitalization or death and to guide public health policy priorities. The excess hospitalization estimates for pneumonia and influenza in our study reflect the considerable effect of influenza in Singapore, particularly among the rapidly aging population.
